# Executive Functioning in Obesity, Food Addiction, and Binge-Eating Disorder

**DOI:** 10.3390/nu11010054

**Published:** 2018-12-28

**Authors:** Marie Blume, Ricarda Schmidt, Anja Hilbert

**Affiliations:** Integrated Research and Treatment Center Adiposity Diseases, Departments of Medical Psychology and Medical Sociology and Psychosomatic Medicine and Psychotherapy, University of Leipzig Medical Center, 04103 Leipzig, Germany; ricarda.schmidt@medizin.uni-leipzig.de (R.S.); anja.hilbert@medizin.uni-leipzig.de (A.H.)

**Keywords:** executive function, obesity, binge-eating disorder, food addiction, addictive-like eating

## Abstract

This study aimed to investigate food addiction (FA) and binge-eating disorder (BED) in their association to executive dysfunctions in adults with obesity. Data on response inhibition, attention, decision-making, and impulsivity were derived from four groups of adults with obesity: obesity and FA (*n* = 23), obesity and BED (*n* = 19), obesity and FA plus BED (FA/BED, *n* = 23), and a body mass index-, age-, and sex-stratified control group of otherwise healthy individuals with obesity (*n* = 23, OB), using established computerized neuropsychological tasks. Overall, there were few group differences in neuropsychological profiles. Individuals of the FA group did not differ from the OB group regarding executive functioning. Individuals with BED presented with significantly higher variability in their reaction times and a deficient processing of feedback for performance improvement compared to individuals of the OB group. Strikingly, individuals with FA/BED did not present neuropsychological impairments, but higher levels of depression than all other groups. The results indicated the presence of a BED-specific neuropsychological profile in the obesity spectrum. The additional trait FA was not related to altered executive functioning compared to the OB or BED groups. Future research is needed to discriminate FA and BED further using food-specific tasks.

## 1. Introduction

Obesity, defined as an excessive accumulation of body fat (body mass index (BMI) ≥ 30.0 kg/m^2^) [1], presents one of the most prevalent health conditions in the Western world [2]. During the last decade, research on the causal and maintaining factors of obesity has focused on executive functioning and neural characteristics [3,4,5]. Executive functions represent a range of higher cognitive capacities enabling goal-directed behavior including inhibition, cognitive flexibility, planning, or decision-making [6,7]. Importantly, impairments in general executive functions in individuals with obesity, for example, reduced inhibition and planning, were linked to unsuccessful weight-loss related behavior, such as poor dietary quality and less success in weight loss therapies [8,9]. In order to provide targeted treatment options for executive dysfunctions, the specification of neuropsychological profiles in the obesity spectrum is warranted [10].

In this context, a recent review showed substantial similarities between executive functions in obesity and substance use disorder, including increased impulsive decision-making and an attentional bias towards disorder-related stimuli in both conditions [11]. In addition to these neuropsychological abnormalities, several neuroimaging studies documented a considerable overlap between obesity and substance use disorder regarding functional alterations in frontal brain regions and meso-corticolimbic circuits, which are involved in reward processing and decision-making [11,12,13].

Recent research hypothesized that certain foods, especially high-fat and high-sugar foods, elicit addictive-like behavioral responses in vulnerable individuals characterized by high impulsivity and reward sensitivity through the activation of reward-related brain circuits [12,14,15,16]. This so-called food addiction (FA) hypothesis has been controversially discussed [15,17,18,19,20], since certain aspects of substance use symptomatology, including symptoms of withdrawal or tolerance, have not been demonstrated regarding food [21]. FA is mainly operationalized as a trait based on a self-report questionnaire to assess food-related addictive behavior, the *Yale Food Addiction Scale 2.0* (YFAS 2.0) [22]. Although FA was found in individuals across the weight range, it was often described in the context of obesity, with highly varying prevalence of 6.7–56.8% in samples of treatment-seeking individuals who were overweight or obese [14]. Two recent studies specifically investigated alterations in executive functioning in individuals with obesity and trait FA, showing that higher versus lower FA severity in obesity was related to poorer decision-making, greater attentional impairments [23], significantly poorer inhibition and cognitive flexibility assessed via self-report questionnaires and a computerized neuropsychological test battery [24]. Individuals with obesity and trait FA, may therefore present with a specific neuropsychological profile in the obesity spectrum.

Likewise, the clinical eating disorder diagnosis of binge-eating disorder (BED) [25] has been associated with addictive-like eating behavior [26,27]. According to the 5th edition of the Diagnostic and Statistical Manual of Mental Disorders (DSM-5) [25], the core feature of BED is represented by recurrent episodes of objective binge eating, which are characterized by a feeling of loss of control over eating while consuming an unambiguously large amount of food in a discrete period of time. BED is not associated with regular inappropriate compensatory behaviors, such as vomiting or excessive exercising [25]. Among treatment-seeking individuals with overweight and obesity, 23.9% presented with comorbid BED [28]. Individuals with BED were repeatedly found to show impairments in executive functioning exceeding those observed in obesity only, including increased impulsivity, reward sensitivity, rash spontaneous behavior, risky decision-making, and reduced inhibition [29,30,31,32,33,34]. These alterations were found in general executive functioning tasks [30,35,36] as well as in food-related tasks [30,37,38], strengthening the assumption of a specific neuropsychological profile of individuals with obesity and comorbid BED.

Even though trait FA and the diagnosis of BED have a significant number of behavioral symptoms in common (e.g., consumption of large amounts of food, loss of control, continued use despite negative consequences, cravings) and show a comorbidity of 57.0–72.2% in treatment-seeking and population-based samples with obesity [39], no study has yet compared the neuropsychological profiles of individuals with obesity presenting with or without FA and BED.

In the context of executive functions in obesity as described above, we therefore aimed to investigate general executive functions, specifically decision-making, reward sensitivity, cognitive flexibility, inhibition, and cognitive control in four distinct groups of individuals with obesity: those with FA, BED, FA plus BED (FA/BED), and a control group of otherwise healthy individuals with obesity (OB) stratified to the other groups based on age, sex, and BMI. Among the four groups, we assumed that individuals with OB would show the highest levels of executive functioning. For those with FA, we particularly expected poorer decision-making, cognitive control, inhibition, and flexibility than for OB. Furthermore, we assumed that individuals with BED would perform significantly worse than individuals with OB in tasks investigating impulsivity, reward sensitivity, and inhibition. Due to a lack of evidence, no evidence-based hypothesis was derived regarding possible differences on executive functioning between individuals with trait FA and those with BED. However, based on a recent finding that individuals with FA plus BED displayed greater eating disorder and general psychopathology than individuals with BED only [40], we suggested cumulative adverse effects on executive functioning. Thus, individuals with FA/BED were expected to show lower levels of executive functioning than individuals with OB, FA, and BED only.

## 2. Materials and Methods

### 2.1. Participants

Adults with obesity were recruited at the time of admission of two outpatient brain-directed psychological treatment studies at the Integrated Research and Treatment Center Adiposity Diseases in Leipzig, Germany (Cognitive remediation therapy for adults with obesity [41]; electroencephalographic neurofeedback training for adults with BED [42]), and were tested prior to the beginning of either intervention. Inclusion and exclusion criteria for both studies were similar. Relatedly, participants included in this study were required to be between 18 and 60 years of age, present with a BMI ≥ 35.0 kg/m^2^, display sufficient German language skills, and provide informed consent. Exclusion criteria were (1) serious somatic conditions (e.g., neurological disorders, stroke, head injury); (2) serious mental conditions (e.g., psychotic disorder, suicidality, substance use disorder, developmental or intellectual disability); (3) physical, mental, or other inability to participate in assessments (e.g., impediment in hearing, vision, or language); (4) previous or planned bariatric surgery; (5) use of medication that impacts weight or executive functioning (e.g., antipsychotics, sedatives, hypnotics); (6) current psychotherapy regarding weight or eating behavior; (7) current participation in other interventional studies; (8) lack of compliance; and (9) pregnancy or lactation. Individuals eligible for the OB group were required not to display any FA symptoms according to the YFAS 2.0 [22,43] and not to report any objective binge-eating episode during the last three months as determined via clinical interview [44,45]. Individuals eligible for the FA group needed to present with trait FA, according to the YFAS 2.0 [22,43], and were required not to report any objective binge-eating episode during the last three months as determined via clinical interview [44,45]. Individuals eligible for the BED group were required to present with the DSM-5 diagnosis of BED assessed with a clinical interview [44,45] and not to present with trait FA according to the YFAS 2.0 [22,43]. Individuals eligible for the FA/BED group had to present with trait FA, according to the YFAS 2.0 [22,43], and with the DSM-5 diagnosis of BED assessed with a clinical interview [44,45].

### 2.2. Ethics and Procedure

Both studies were approved by the Ethical Committee of Leipzig University Medical Center (256-15-13072015, 143-15-20042015) and carried out in accordance with the Declaration of Helsinki. Within the two mentioned treatment studies, all participants were screened for eligibility using a standardized telephone interview. All eligible participants were invited to a baseline in-person diagnostic session. At the beginning of the diagnostic session, informed consent was obtained, followed by a series of neuropsychological tasks (see below). BMI was calculated based on participants’ weight (in kg) and height (in cm) which were objectively measured using calibrated instruments at the end of the diagnostic session. The diagnostic sessions were standardized and conducted by trained staff. All participants were asked to refrain from eating at least 2 h prior to testing, in order to avoid any uncontrolled confounding effects of hunger or satiety on executive functioning.

### 2.3. Sample

Between November 2015 and April 2018, a total of *n* = 1082 individuals registered with an interest in one of the two brain-directed treatment studies, of whom a total of *n* = 258 were eligible and included in one of these brain-directed treatment studies. All *n* = 258 individuals participated in the baseline diagnostic session. For the present study, a convenience sample out of this study population was used, consisting of *n* = 23 individuals with trait FA based on the YFAS 2.0 [22,43], *n* = 19 with the DSM-5 diagnosis of BED based on clinical interview [44,45], and *n* = 23 individuals characterized by trait FA and BED (FA/BED). Out of *n* = 85 individuals eligible for the OB group, a total of *n* = 23 otherwise healthy individuals with obesity were stratified to the mean scores of the other groups regarding age, BMI, sex, and education.

### 2.4. Measures

#### 2.4.1. Eating Disorder Examination

The diagnosis of BED according to DSM-5 [25] was derived from the diagnostic items of the validated German version of the Eating Disorder Examination interview (EDE 17.0D) [44,45]. According to DSM-5, the following severity levels of BED were determined: mild (1–3 objective binge-eating episodes per week), moderate (4–7 objective binge-eating episodes per week), severe (8–13 objective binge-eating episodes per week), and extreme (≥14 objective binge-eating episodes per week). The EDE has high interrater reliability (*r* ≥ 0.90) [46].

#### 2.4.2. Yale Food Addiction Scale

The validated German version of the 35-item self-report Yale Food Addiction Scale 2.0 (YFAS 2.0; α = 0.914) [22,43] was used to determine FA. Participants were instructed to indicate how often they experienced addictive-like behavior towards food over the last year, with responses ranging from 0 (never) to 7 (every day). A total score was calculated by adding up all DSM-5 symptoms for addiction, ranging from 0 to 11. Scores ≥ 2 in combination with clinically significant distress indicate the presence of FA, with higher scores representing more severe FA: mild (2–3 symptom scores), moderate (4–5 symptom scores), and severe (≥6 symptom scores).

#### 2.4.3. Eating Disorder Examination-Questionnaire

The validated German version of the short 8-item version of the Eating Disorder Examination-Questionnaire (EDE-Q8; α = 0.766) [47] was used to evaluate participants’ global eating disorder psychopathology. A mean score, ranging from 0 to 6, was calculated, with higher scores indicating greater eating disorder psychopathology.

#### 2.4.4. Patient Health Questionnaire-Depression Scale

The validated German version of the 9-item Patient Health Questionnaire-Depression Scale (PHQ-9; α = 0.813) [48,49] was administered to assess the level of depression based on the DSM-5 criteria for depression. Items were scored on a 0 (not at all) to 3 (almost every day) scale. Participants’ sum scores were computed, ranging from 0 to 27, with higher scores representing more severe depression. Scores < 5 indicate no depressive symptoms, scores between 5 and 10 a mild depression, and scores > 10 a major depression [48,49].

### 2.5. Neuropsychological Assessment

#### 2.5.1. Iowa Gambling Task

The computerized version of the Iowa Gambling Task (IGT) [50] was used to assess decision-making under uncertainty and complexity. The IGT requires participants to choose 100 times from four possible card decks (A, B, C, and D), aiming at winning the highest possible amount of virtual money. Each card deck contains profits and losses of different amounts. In the long term, choosing from card decks A and B will result in overall long-term loss (disadvantageous decks), whereas choosing cards from card decks C and D will result in overall long-term gain (advantageous decks). Performance in the IGT was determined by the total net score, calculated by subtracting the total number of disadvantageous choices from the total number of advantageous choices. Additionally, a learning effect across the net score of the five consecutive blocks of 20 cards was considered.

#### 2.5.2. Delay Discounting Task

The computerized version of the Delay Discounting Task (DDT) [51], provided by the test software Millisecond [52] was used to determine the individual discounting rate of delayed rewards, which represents a measure of impulsive decision-making [51]. In this paradigm participants were instructed to choose between a standard amount of money (10 EUR) with different temporal delays (0, 2, 30, 180, and 365 days) or a variable amount (0–10 EUR) with no delay, until an indifference point for each delay was found or until the maximum number of trials (30) has been run for each delay. Five indifference points were calculated, where the immediate reward and delayed reward were equal in terms of subjective value. To determine performance in the DDT, the five indifference points were used to calculate the area under the curve (AUC) [53]. The AUC may range from 0 to 1 with greater AUC values being associated with less discounting of delayed rewards, i.e., less impulsivity [53].

#### 2.5.3. Go/No Go

The computerized version of the visual Go/No Go paradigm [54], provided by the Vienna Test System [55], was used to assess inhibitory control. The visual Go/No Go paradigm required participants to determine whether a stimulus required a reaction or an inhibition. Therefore, 202 triangles were shown on the screen, which required a fast response, while a total of 48 circles appeared randomly in-between, indicating that no response should be given. Performance in the Go/No Go paradigm was determined by the number of commission errors (false positive responses towards a No Go trial), with more commission errors suggesting diminished inhibitory control [56].

#### 2.5.4. Wisconsin Card Sorting Test

The computerized version of the Wisconsin Card Sorting Test (WCST) [57] was used as a measure of cognitive flexibility. The WCST required participants to sort each of maximally 128 cards according to three possible principles or rules (e.g., shape, color, or number), which were unknown to the participant, but would become apparent via feedback during the trials. After 10 consecutively and correctly sorted cards the rule changed suddenly and without warning. The participant then had to generate solutions and use the feedback to adapt to the new rule. Performance on the WCST was measured with the percentage of perseverative errors, which represent a tendency to perseverate on the previous rule, and the learning to learn score, which represents the average growth of conceptual efficiency during the test (average difference in percent errors between successive categories). The learning to learn score can only be calculated if participants have completed at least three categories or completed two categories and attempted a third. The learning to learn score can obtain positive and negative values, with positive scores indicating enhanced efficiency across consecutive categories, presumably because of learning [57].

#### 2.5.5. Alertness

Alertness and the variability in early attentional processes was assessed with a computerized perception and attention functions battery (WAFA) [58], provided by the Vienna Test System [55]. To assess visual intrinsic alertness, participants were required to look at a fixation cross at the center of the screen and press a button as fast as possible when a target stimulus, a circle, appeared. For the assessment of phasic alertness, an auditory cue appeared shortly before the visual stimulus, preparing the participant for next execution of the reaction. Performance in intrinsic and phasic alertness was reported as log-transformed mean reaction time, with higher scores indicating longer reaction times and therefore less alertness [58]. Variability in performance in intrinsic and phasic alertness was reported as a measure of dispersion, with higher scores indicating greater variability in intrinsic or phasic alertness.

### 2.6. Statistical Procedure

Statistical analyses were performed using IBM SPSS Statistics version 24 (IBM Corp. Released 2016. IBM SPSS Statistics for Windows, Version 24.0. Armonk, NY: IBM Corp). All tests were two-tailed and considered significant when *p* values were <0.05. Since the domains of executive functioning are distinct and based on single measures, adjusting for multiple testing was not necessary, except for the computerized alertness task (WAFA) where four outcome measures were used. The *p* value for testing group differences in the WAFA was therefore adjusted to *p* = 0.05/4 = 0.0125. For the comparison of groups regarding socio-demographic and clinical variables, univariate analyses of variance (ANOVAs) were calculated for continuous variables and Chi-square tests (Χ^2^) for categorical variables. The prerequisites for performing the tests (normal distribution and homogeneity of variance) were tested with the Shapiro-Wilk and Levene tests. When prerequisites were not met, Welch ANOVA was used, because of its robustness against violations of the assumption of homogeneity of variances [59]. In case of significance and homogeneity of variances, the Bonferroni post-hoc test was used to determine pair-wise differences; in case of violation of homogeneity of variances the Games-Howell post-hoc test was used. For Welch ANOVA, estimated omega squared (est. ω^2^) was calculated for which ω^2^ ≥ 0.01 is considered a small effect, ω^2^ ≥ 0.06 a moderate, and ω^2^ ≥ 0.14 a large effect [60]. For mean differences in post-hoc tests Cohen’s *d* was calculated, with *d* ≥ 0.2 being considered a small effect, *d* ≥ 0.5 a moderate, and *d* ≥ 0.8 a large effect [61]. In the present study, a power of ≥ 0.75 was observed to detect moderate and ≥ 0.95 to detect large group differences in ANOVAs (two-tailed *p* < 0.05).

## 3. Results

### 3.1. Sample

As depicted in Table 1, groups did not differ in socio-demographic characteristics. Significant differences between groups were found in eating disorder psychopathology and depression. For eating disorder psychopathology, post-hoc tests did not reach significance. The FA/BED group showed significantly more depressive symptoms compared to the OB group (5.17, 95% Confidence interval (CI) (1.86, 8.49), *p* ≤ 0.001). Significantly more individuals of the FA/BED group presented with severe FA compared to the FA group, where there were more cases of mild FA. The FA/BED group did not differ significantly regarding BED severity from the BED group.

### 3.2. Neuropsychological Assessment

**Figure 1:**
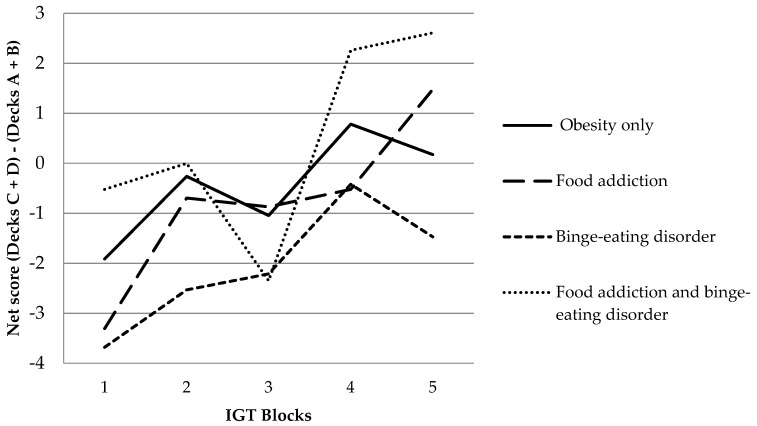
Iowa Gambling Task (IGT) learning effect across five consecutive blocks.

In the WAFA, groups differed significantly in the variability to react to a cued target (measure of dispersion phasic alertness), see Table 2, with individuals with BED showing significantly more variability in reaction times than individuals with OB (0.88, 95% CI [0.13, 1.63], *p* = 0.018, *d* = 1.12). Significant group differences were furthermore observed in the growth of conceptual efficiency in the WCST (learning to learn score), indicating that individuals with BED showed significantly less growth of conceptual efficiency in learning compared to individuals with OB (−3.47, 95% CI [−6.92, −0.01], *p* = 0.049, *d* = −0.85). Regarding decision-making, all groups improved their net score over the five consecutive blocks of the IGT, as depicted in Figure 1. Although descriptive data of the IGT net score seem to differ substantially, no significant group differences were found and only a small effect size was observed (see Table 2), suggesting that trait FA and BED diagnosis were not associated with altered decision-making in individuals with obesity. In addition, groups did not differ in their mean reaction time (intrinsic and phasic) and variability to react to un-cued visual targets (measure of dispersion intrinsic alertness) in the WAFA, the amount of perseverative errors in the WCST, and the amount of commission errors in the Go/No Go task (see Table 2), indicating that trait FA and BED were not associated with altered attention, cognitive flexibility, and inhibition in individuals with obesity.

## 4. Discussion

The present study aimed at investigating the associations of trait FA and the DSM-5 diagnosis of BED regarding executive functions in adults with obesity. It was found that individuals with BED showed lower executive functioning, characterized by a higher variability in reaction times and less feedback integration, compared to otherwise healthy individuals with obesity. Contrary to expectations, there were no differences in executive functioning among individuals with FA, FA/BED, and obesity only. The results thus suggest a BED-specific, but not a FA-specific, neuropsychological profile in individuals with obesity. The combined presentation of trait FA and BED was not associated with greater impairments in executive functioning compared to their single presentations.

The fact that individuals with obesity and BED displayed a significantly higher variability in their reaction times compared to the OB group is similar to findings showing a high variability in reaction times in ADHD [62]. ADHD is highly comorbid to obesity and BED and is associated with comparable neuronal processes [62,63,64]. In ADHD, it is usually believed that the variability of response times reflects occasional gaps in attention [62], which could be related to high distractibility, making an attentional shift and the detection of task-irrelevant stimuli, for example, food cues, more likely. Another result was the significant and medium-sized difference between the BED and the OB group in the learning to learn score in the WCST, in accordance with recent research [37]. The negative learning to learn score indicated that individuals with BED became less rather than more efficient on consecutive trials of the WCST [57]. Hypothetically, this could be caused by an inability to appropriately integrate the feedback in order to improve performance, as previously evidenced in BED [29]. Transferred into everyday life, the deficient processing of feedback to improve performance could be related to a lack of feedback integration in daily learning, such as feedback given by one’s own body after an objective binge-eating episode in order to prevent such an episode from occurring again, leaving an individual without control [65]. In this context, it is worth noting that the BED group did not display heightened depressive symptoms compared to the OB and FA group. Observed differences between the BED and OB group can therefore not be attributed to increased general psychopathology, but were related to BED diagnosis.

Contrary to the two previous studies investigating executive functions in individuals with trait FA [23,24], demonstrating that higher versus lower FA severity in obesity was related to poorer decision-making, inhibition, and cognitive flexibility, no other performance score reached significance in the present study. The hypothesis that individuals with obesity and FA may present with a specific neuropsychological profile in the obesity spectrum was therefore not supported in this clinical treatment-seeking sample. It is of note, however, that the FA group mostly presented with mild and moderate FA symptomatology only, allowing to speculate that FA severity was not high enough to be accompanied by cognitive impairments in this sample. On the other hand, this result could also reflect the assumption that BED, as a clinical eating disorder diagnosis, is associated with more cognitive impairments than subjective trait FA.

The hypothesis of cumulative effects on executive dysfunctions in individuals with FA plus BED was not confirmed in the present study, since the FA/BED group did not display lower performance compared to the OB, FA, or BED group in any of the tasks under investigation. Notably, the FA/BED group reported considerably more depressive symptoms compared to the OB group, with an average in the range of a major depression [48,49], and thus contradicts neuropsychological evidence of impaired executive functioning in individuals with depression [66]. However, the underlying mechanisms of this finding cannot currently be identified. Therefore, this result needs replication in larger, independent samples of individuals with FA/BED.

### 4.1. Methodological Considerations

When interpreting the results several strength and limitations have to be mentioned. First, using well-established measures, the diagnosis of BED was derived from an expert interview, the EDE 17.0D [44,45], and trait FA was assessed via the established self-questionnaire YFAS 2.0 [22,43]. Second, the OB group was stratified to the other groups and groups were mutually exclusive regarding trait FA and objective binge-eating episodes. Third, executive functioning was measured with established, standardized, and computerized tasks. Critically, the relatively small group sizes have to be mentioned, as only medium-to-large-sized effects could be detected with adequate power. Additionally, there might be an assessment bias across diagnostic groups, as FA was assessed with a self-administered questionnaire, while BED diagnosis was made by clinical expert interview, suggesting that trait FA and BED mirror different levels of clinical severity. Furthermore, no food-specific tasks were used. Various studies have shown a stronger association between executive functions in food-related tasks in relation to binge-eating behavior [30,38]. Lastly it is important to note that the design of this study was cross-sectional; therefore, causal conclusions about the direction of observed effects are not indicated.

### 4.2. Implications and Future Directions

The current study identified greater executive dysfunctions in individuals with obesity and comorbid BED compared to those with obesity only. Clinically, the assessment of eating disorder symptomatology, specifically a diagnosis of BED, seems therefore indicated in individuals with obesity. In these individuals, an increase in neuropsychological impairments is likely to interfere with standard treatment options [8,9] and may thus constitute a specific target for intervention, for example, via cognitive remediation therapy [41]. Based on this study’s results, an in-depth investigation in the variability of reaction times (alertness tasks) is indicated, especially considering the effects of food stimuli, as current research points to an increased attentional bias towards food cues in individuals with BED e.g., [66,67].

Regarding trait FA, this study revealed no impairments in executive functioning other than a tendency towards higher choice-impulsivity, which could be related to the high proportion of individuals characterized by low FA severity in the FA group. Studies investigating executive functions in individuals with obesity and more severe FA are therefore needed. Alternatively, it could be argued that similarities in executive functioning between individuals with obesity plus trait FA and individuals with substance use disorder as recently shown [12] only manifest on a neurobiological [12] and not on a neuropsychological level. To further differentiate these aspects of addictive-like eating behavior, future research is warranted to specifically investigate neuronal and neuropsychological overlaps and distinctive features between trait FA and substance use disorder, especially in the context of disorder-related stimuli (e.g., food stimuli).

It was surprising that the observed large differences in attentional variability and conceptual learning between the BED and OB group did not manifest in the FA/BED group, particularly as this group presented as the most impaired group concerning mental health, with more depressive symptoms than the OB group. The assessment of trait FA in individuals with versus without BED may therefore be helpful to determine identify individuals at risk for greater mental health impairment and in need of more intensive treatment. Further research into the neuropsychological characteristics of individuals with FA plus BED is however warranted, preferably using larger samples and a longitudinal study design in order to evaluate the effects of trait FA plus BED on weight trajectories and treatment outcome in individuals with obesity.

## Figures and Tables

**Table 1:** Sociodemographic characteristics.

Variable	Obesity Only *n* = 23	Food Addiction *n* = 23	Binge-Eating Disorder *n* = 19	Food Addiction Plus Binge-Eating Disorder *n* = 23	Test			Effect Size
	*M (SD)*	*M (SD)*	*M (SD)*	*M (SD)*	*F*	*df*	*p*	*Est.* ω^2^
Age (years)	40.48 (10.85)	43.39 (10.35)	38.84 (9.43)	37.53 (10.12)	1.38	3, 84	0.254	0.013
Body mass index (kg/m^2^)	42.84 (4.76)	44.14 (6.59)	41.92 (5.25)	42.23 (6.13)	0.64	3, 84	0.589	−0.012
	*n*	*n*	*n*	*n*	Χ^2^	*df*	*p*	
Sex (female/male)	17/6	18/5	14/5	16/7	0.45	3	0.930	
Education (low/middle/high) ^1^	3/11/9	3/16/4	1/7/11	4/10/9	8.50	6	0.203	
	*M (SD)*	*M (SD)*	*M (SD)*	*M (SD)*	*F*	*df*	*p*	*Est.* ω^2^
Eating disorder psychopathology (EDE-Q8)	3.59 (0.98) ^a^	4.20 (0.80) ^a^	3.55 (1.03) ^a^	4.36 (1.20) ^a^	3.92	3, 84	0.011	0.091
Depression (PHQ-D)	5.00 (3.47) ^a^	7.71 (3.80) ^a, b^	7.99 (5.27) ^a, b^	10.17 (4.10) ^b^	5.98	3, 84	0.001	0.145
	*n*	*n*	*n*	*n*	Χ^2^	*df*	*p*	
FA severity (mild/moderate/severe)	-	9/10/4	-	0/6/17	18.05	2	<0.001	
BED severity (mild/moderate/severe)	-	-	14/5/0	16/4/3	2.89	2	0.236	

Note. M: mean, SD: standard deviation, df: degrees of freedom, FA: food addiction, BED: binge-eating disorder, BMI: body mass index, PHQ-D: Patient Health Questionnaire-Depression Scale, EDE-Q8: short version of the Eating Disorder Examination-Questionnaire ^a,b^ Different superscripts denote significant differences in post-hoc comparisons with Bonferroni corrections. ^1^ In Germany, secondary school is subdivided into three tracks: lower ≤ 9 years of education, middle = 10 years of education, high ≥ 12 years of education.

**Table 2:** Performance on neuropsychological assessments.

Variable		Obesity Only *n* = 23	Food Addiction *n* = 23	Binge-Eating-Disorder *n* = 19	Food Addiction plus Binge-Eating Disorder *n* = 23	Welch ANOVA			Effect Size
		*M (SD)*	*M (SD)*	*M (SD)*	*M (SD)*	*F*	*df*	*p*	*Est*. ω^2^
IGT	Net score	−2.26 (32.78)	−3.91 (32.18)	−10.32 (39.36)	2.00 (35.17)	0.37	3, 46	0.773	−0.022
DDT	Area under the curve	0.47 (0.32)	0.31 (0.26)	0.54 (0.26)	0.46 (0.24)	2.80	3, 46	0.050	0.058
Go/No Go	Commission errors	11.61 (5.98)	11.39 (7.88)	9.74 (6.06)	12.52 (7.22)	0.64	3, 46	0.591	−0.012
WCST	Perseverative errors	17.70 (6.67)	17.40 (8.38)	14.31 (4.75)	18.38 (8.28)	2.02	3, 46	0.125	0.034
	Learning to learn ^1^	2.01 (4.22) ^a^	0.88 (6.60) ^a, b^	−1.45 (3.90) ^b^	3.70 (10.08) ^a, b^	2.94	3, 40	0.045	0.069
WAFA	Mean reaction time intrinsic in ms	254.22 (45.69)	264.61 (45.02)	265.37 (43.22)	266.00 (72.05)	0.30	3, 46	0.824	−0.024
	Mean reaction time phasic in ms	253.78 (57.09)	242.04 (61.98)	223.63 (88.57)	249.00 (95.92)	0.56	3, 45	0.642	−0.015
	Measure of dispersion intrinsic	1.18 (0.67)	1.24 (0.11)	1.18 (0.06)	1.28 (0.39)	2.53	3, 45	0.069	0.050
	Measure of dispersion phasic	1.41 (0.18) ^a^	1.56 (0.50) ^a, b^	2.29 (1.15) ^b^	1.79 (1.02) ^a, b^	4.80	3, 37	0.006	0.115

Note. M: mean, SD: standard deviation, df: degrees of freedom, IGT: Iowa Gambling Task, DDT: Delay Discounting Task, WAFA: Perception and attention functions battery alertness, WCST: Wisconsin Card Sorting Task, ms: milliseconds. ^a,b^ Different superscripts denote significant differences in post-hoc comparisons with Games-Howell corrections. ^1^ Sample size for learning to learn score *n* = 79 (*n* = 22 obesity only, *n* = 19 food addiction, *n* = 18 binge-eating disorder, *n* = 20 food addiction plus binge-eating disorder).

## References

[1] World Health Organization [WHO] Obesity and Overweight: Fact Sheet. http://www.who.int/mediacentre/factsheets/fs311/en/.

[2] NCD Risk Factor Collaboration (NCD-RisC) (2017). Worldwide trends in body-mass index, underweight, overweight, and obesity from 1975 to 2016: A pooled analysis of 2416 population-based measurement studies in 128·9 million children, adolescents, and adults. Lancet.

[3] Horstmann A. (2017). It wasn’t me; it was my brain-Obesity-associated characteristics of brain circuits governing decision-making. Physiol. Behav..

[4] Makaronidis J.M., Batterham R.L. (2018). Obesity, body weight regulation and the brain: Insights from fMRI. Br. J. Radiol..

[5] Yang Y., Shields G.S., Guo C., Liu Y. (2018). Executive function performance in obesity and overweight individuals: A meta-analysis and review. Neurosci. Biobehav. Rev..

[6] Shallice T. (1988). From Neuropsychology to Mental Structure.

[7] Stuss D.T., Benson D.F. (1986). The Frontal Lobes.

[8] Wyckoff E.P., Evans B.C., Manasse S.M., Butryn M.L., Forman E.M. (2017). Executive functioning and dietary intake: Neurocognitive correlates of fruit, vegetable, and saturated fat intake in adults with obesity. Appetite.

[9] Brockmeyer T., Hamze Sinno M., Skunde M., Wu M., Woehning A., Rudofsky G., Friederich H.-C. (2016). Inhibitory control and hedonic response towards food interactively predict success in a weight loss programme for adults with obesity. Obes. Facts.

[10] Jones A., Hardman C.A., Lawrence N., Field M. (2017). Cognitive training as a potential treatment for overweight and obesity: A critical review of the evidence. Appetite.

[11] Michaud A., Vainik U., Garcia-Garcia I., Dagher A. (2017). Overlapping neural endophenotypes in addiction and obesity. Front. Endocrinol..

[12] Lindgren E., Gray K., Miller G., Tyler R., Wiers C.E., Volkow N.D., Wang G.-J. (2018). Food addiction: A common neurobiological mechanism with drug abuse. Front. Biosci. Landmark Ed..

[13] Jauch-Chara K., Oltmanns K.M. (2014). Obesity—A neuropsychological disease? Systematic review and neuropsychological model. Prog. Neurobiol..

[14] Burrows T., Kay-Lambkin F., Pursey K., Skinner J., Dayas C. (2018). Food addiction and associations with mental health symptoms: A systematic review with meta-analysis. J. Hum. Nutr. Diet..

[15] Gordon E.L., Ariel-Donges A.H., Bauman V., Merlo L.J. (2018). What is the evidence for “food addiction?” a systematic review. Nutrients.

[16] Smith D.G., Robbins T.W. (2013). The neurobiological underpinnings of obesity and binge eating: A rationale for adopting the food addiction model. Biol. Psychiatry.

[17] Davis C. (2017). A commentary on the associations among “food addiction”, binge eating disorder, and obesity: Overlapping conditions with idiosyncratic clinical features. Appetite.

[18] Gearhardt A.N., Corbin W.R., Brownell K.D. (2009). Preliminary validation of the yale food addiction scale. Appetite.

[19] Lacroix E., Tavares H., von Ranson K.M. (2018). Moving beyond the “eating addiction” versus “food addiction” debate: Comment on Schulte et al. (2017). Appetite.

[20] Schulte E.M., Potenza M.N., Gearhardt A.N. (2017). A commentary on the “eating addiction” versus “food addiction” perspectives on addictive-like food consumption. Appetite.

[21] Hilbert A. (2002). Binge-Eating Disorder.

[22] Gearhardt A.N., Corbin W.R., Brownell K.D. (2016). Development of the yale food addiction scale version 2.0. Psychol. Addict. Behav..

[23] American Psychiatric Association (2013). DSM-5: Diagnostic and Statistical Manual of Mental Disorders.

[24] Steward T., Mestre-Bach G., Vintró-Alcaraz C., Lozano-Madrid M., Agüera Z., Fernández-Formoso J.A., Granero R., Jiménez-Murcia S., Vilarrasa N., García-Ruiz-de-Gordejuela A. (2018). Food addiction and impaired executive functions in women with obesity. Eur. Eat. Disord. Rev..

[25] Rodrigue C., Ouellette A.-S., Lemieux S., Tchernof A., Biertho L., Bégin C. (2018). Executive functioning and psychological symptoms in food addiction: A study among individuals with severe obesity. Eat. Weight Disord..

[26] Cassin S.E., von Ranson K.M. (2007). Is binge eating experienced as an addiction?. Appetite.

[27] Davis C., Mackew L., Levitan R.D., Kaplan A.S., Carter J.C., Kennedy J.L. (2017). Binge eating disorder (BED) in relation to addictive behaviors and personality risk factors. Front. Psychol..

[28] Ricca V., Castellini G., Lo Sauro C., Ravaldi C., Lapi F., Mannucci E., Rotella C.M., Faravelli C. (2009). Correlations between binge eating and emotional eating in a sample of overweight subjects. Appetite.

[29] Córdova M.E., Schiavon C.C., Busnello F.M., Reppold C.T. (2017). Nutritional and neuropsychological profile of the executive functions on binge eating disorder in obese adults. Nutr. Hosp..

[30] Giel K.E., Teufel M., Junne F., Zipfel S., Schag K. (2017). Food-related impulsivity in obesity and binge eating disorder—A Systematic update of the evidence. Nutrients.

[31] Kollei I., Rustemeier M., Schroeder S., Jongen S., Herpertz S., Loeber S. (2018). Cognitive control functions in individuals with obesity with and without binge-eating disorder. Int. J. Eat. Disord..

[32] Leehr E.J., Schag K., Dresler T., Grosse-Wentrup M., Hautzinger M., Fallgatter A.J., Zipfel S., Giel K.E., Ehlis A.-C. (2018). Food specific inhibitory control under negative mood in binge-eating disorder: Evidence from a multimethod approach. Int. J. Eat. Disord..

[33] Manasse S.M., Forman E.M., Ruocco A.C., Butryn M.L., Juarascio A.S., Fitzpatrick K.K. (2015). Do executive functioning deficits underpin binge eating disorder? A comparison of overweight women with and without binge eating pathology. Int. J. Eat. Disord..

[34] Svaldi J., Naumann E., Biehl S., Schmitz F. (2015). Impaired early-response inhibition in overweight females with and without binge eating disorder. PLoS ONE.

[35] Kessler R.M., Hutson P.H., Herman B.K., Potenza M.N. (2016). The neurobiological basis of binge-eating disorder. Neurosci. Biobehav. Rev..

[36] Schag K., Schönleber J., Teufel M., Zipfel S., Giel K.E. (2013). Food-related impulsivity in obesity and binge eating disorder—A systematic review: Food-related impulsivity. Obes. Rev..

[37] Balodis I.M., Grilo C.M., Potenza M.N. (2015). Neurobiological features of binge eating disorder. CNS Spectr..

[38] Kittel R., Brauhardt A., Hilbert A. (2015). Cognitive and emotional functioning in binge-eating disorder: A systematic review. Int. J. Eat. Disord..

[39] Davis C., Curtis C., Levitan R.D., Carter J.C., Kaplan A.S., Kennedy J.L. (2011). Evidence that “food addiction” is a valid phenotype of obesity. Appetite.

[40] Gearhardt A.N., White M.A., Masheb R.M., Morgan P.T., Crosby R.D., Grilo C.M. (2012). An examination of the food addiction construct in obese patients with binge eating disorder. Int. J. Eat. Disord..

[41] Hilbert A., Blume M., Petroff D., Neuhaus P., Smith E., Hay P.J., Hübner C. (2018). Group cognitive remediation therapy for adults with obesity prior to behavioural weight loss treatment: Study protocol for a randomised controlled superiority study (CRT study). BMJ Open.

[42] Blume M., Schmidt R., Schmidt J., Martin A., Hilbert A. German Clinical Trials Register: EEG Neurofeedback for Adults with Binge-Eating Disorder. https://www.drks.de/drks_web/navigate.do?navigationId=trial.HTML&TRIAL_ID=DRKS00010496.

[43] Meule A., Müller A., Gearhardt A.N., Blechert J. (2017). German version of the Yale Food Addiction Scale 2.0: Prevalence and correlates of “food addiction” in students and obese individuals. Appetite.

[44] Fairburn C.G., Cooper Z., O’Connor M. (2014). Eating Disorder Examination (17.0D).

[45] Hilbert A., Tuschen-Caffier B. (2016). Eating Disorder Examination. Deutschsprachige Übersetzung.

[46] Berg K.C., Peterson C.B., Frazier P., Crow S.J. (2012). Psychometric evaluation of the eating disorder examination and eating disorder examination-questionnaire: A systematic review of the literature. Int. J. Eat. Disord..

[47] Kliem S., Mößle T., Zenger M., Strauß B., Brähler E., Hilbert A. (2016). The eating disorder examination-questionnaire 8: A brief measure of eating disorder psychopathology (EDE-Q8). Int. J. Eat. Disord..

[48] Gräfe K., Zipfel S., Herzog W., Löwe B. (2004). Screening psychischer Störungen mit dem “Gesundheitsfragebogen für Patienten (PHQ-D)”. Diagnostica.

[49] Spitzer R.L., Kroenke K., Williams J.B. (1999). Validation and utility of a self-report version of PRIME-MD: The PHQ primary care study. Primary Care Evaluation of Mental Disorders. Patient Health Questionnaire. JAMA.

[50] Bechara A., Damasio A.R., Damasio H., Anderson S.W. (1994). Insensitivity to future consequences following damage to human prefrontal cortex. Cognition.

[51] Richards J.B., Zhang L., Mitchell S.H., de Wit H. (1999). Delay or probability discounting in a model of impulsive behavior: Effect of alcohol. J. Exp. Anal. Behav..

[52] Millisecond (2015). Inquisit (Version 4).

[53] Myerson J., Green L., Warusawitharana M. (2001). Area under the curve as a measure of discounting. J. Exp. Anal. Behav..

[54] Kaiser S., Aschenbrenner S., Pfüller U., Roesch-Ely D., Weisbrod M. (2015). Wiener Testsystem: Response Inhibition.

[55] Schuhfried Gmbh (2015). Vienna Test System.

[56] Lezak M.D., Howieson D.B., Bigler E.D., Tranel D. (2012). Neuropsychological Assessment.

[57] Heaton R.K., Chelune G.J., Talley J.L., Kay G.G., Curtiss G. (1993). Wisconsin Card Sorting Test Manual.

[58] Sturm W. (2015). Wiener Testsystem: Wahrnehmungs- und Aufmerksamkeitsfunktionen.

[59] Lix L.M., Keselman J.C., Keselman H.J. (1996). Consequences of assumption violations revisited: A quantitative review of alternatives to the one-way analysis of variance f test. Rev. Educ. Res..

[60] Keppel G. (1991). Design and Analysis: A Researcher’s Handbook.

[61] Cohen J. (1988). Statistical Power Analysis for the Behavioral Sciences.

[62] Tamm L., Narad M.E., Antonini T.N., O’Brien K.M., Hawk L.W., Epstein J.N. (2012). Reaction time variability in ADHD: A review. Neurotherapeutics.

[63] Cortese S., Bernardina B.D., Mouren M.-C. (2007). Attention-deficit/hyperactivity disorder (adhd) and binge eating. Nutr. Rev..

[64] Voon V. (2015). Cognitive biases in binge eating disorder: The hijacking of decision making. CNS Spectr..

[65] Restivo M.R., McKinnon M.C., Frey B.N., Hall G.B., Syed W., Taylor V.H. (2017). The impact of obesity on neuropsychological functioning in adults with and without major depressive disorder. PLoS ONE.

[66] Schmidt R., Lüthold P., Kittel R., Tetzlaff A., Hilbert A. (2016). Visual attentional bias for food in adolescents with binge-eating disorder. J. Psychiatr. Res..

[67] Sperling I., Baldofski S., Lüthold P., Hilbert A. (2017). Cognitive food processing in binge-eating disorder: An eye-tracking study. Nutrients.

